# Dampening Enthusiasm for Circulating MicroRNA in Breast Cancer

**DOI:** 10.1371/journal.pone.0057841

**Published:** 2013-03-05

**Authors:** Rom S. Leidner, Li Li, Cheryl L. Thompson

**Affiliations:** 1 Department of Medicine, Case Western Reserve University, Cleveland, Ohio, United States of America; 2 Department of Family Medicine and Community Health, Case Western Reserve University, Cleveland, Ohio, United States of America; 3 Department of Epidemiology and Biostatistics, Case Western Reserve University, Cleveland, Ohio, United States of America; 4 Case Comprehensive Cancer Center, Case Western Reserve University, Cleveland, Ohio, United States of America; University of Barcelona, Spain

## Abstract

Genome-wide platforms for high-throughput profiling of circulating miRNA (oligoarray or miR-Seq) offer enormous promise for agnostic discovery of circulating miRNA biomarkers as a pathway for development in breast cancer detection. By harmonizing data from 15 previous reports, we found widespread inconsistencies across prior studies. Whether this arises from differences in study design, such as sample source or profiling platform, is unclear. As a reproducibility experiment, we generated a genome-wide plasma miRNA dataset using the Illumina oligoarray and compared this to a publically available dataset generated using an identical sample size, substrate and profiling platform. Samples from 20 breast cancer patients, 20 mammography-screened controls, as well as 20 breast cancer patients after surgical resection and 10 female lung or colorectal cancer patients were included. After filtering for miRNAs derived from blood cells, and for low abundance miRNAs (non-detectable in over 10% of samples), a set of 522 plasma miRNAs remained, of which 46 were found to be differentially expressed between breast cancer patients and healthy controls (p<0.05), of which only 3 normalized to baseline levels in post-resection cases and were unique to breast cancer vs. lung or colorectal cancer (miR-708*, miR-92b* and miR-568, none previously reported). We were unable to demonstrate reproducibility by various measures between the two datasets. This finding, along with widespread inconsistencies across prior studies, highlight the need for better understanding of factors influencing circulating miRNA levels as prerequisites to progress in this area of translational research.

## Introduction

Breast cancer is the leading cancer in women in the United States, and the third leading cause of cancer deaths among women [1]. While long term survival for localized breast cancer is high (over 95%), five year survival declines sharply with stage (84% for regional involvement and 27% distant spread) [1], underscoring the importance of early detection. Despite widespread use in the US, mammography is not an ideal screening modality [2]. High false positive rates result in unacceptable rates of unnecessary biopsies each year, which in turn, increases health care costs and the anxiety associated with screening. This has led to controversial recommendations to reduced the frequency of screening [3], and many women already perceive mammography as uncomfortable and/or time consuming, influencing their rates of compliance [4], [5]. A blood-based alternative would have a major clinical impact for screening, as well as for monitoring response to treatment and long term surveillance.

MicroRNAs (miRNAs) are short non-coding segments of RNA that bind directly to messenger RNA to suppress translation of target genes [6]. The high level of conservation between species in miRNA coding regions indicates a critical biologic role [6]. In cancer, miRNAs have been shown to be deregulated in tissue specific patterns which uniquely classify every type of tumor studied to date [7], and are disproportionately localized to regions of genomic fragility in cancer [8]. An ensemble of miRNAs are known to be deregulated in breast cancer [7], [9], with specific miRNAs correlated to breast cancer subtype, prognosis, metastasis [10] and treatment resistance [11]. Functional studies have further demonstrated the mechanisms through which these miRNAs are intimately involved in tumor biology of the breast [12]–[16]. In the circulation, miRNAs were recently identified at unexpectedly high levels and found to be the most stable nucleic acid in peripheral blood. This exciting discovery immediately spurred a rush to investigate circulating miRNAs as a novel biomarker for minimally invasive early cancer detection [17], [18]. Since late 2009, ten studies from 9 independent groups in 5 different countries reported circulating miRNA profiling in breast cancer by screening a handful of selected miRNA by qPCR [19]–[28]. Collectively, these selected probe-set studies, profiled 25 candidate miRNAs using this approach, using serum or whole blood samples representing a total of 541 breast cancer cases and 326 healthy controls. The composite was a short list of 16 differential miRNAs, which were significantly altered in the circulation of breast cancer cases (10 up, 6 down). However, only two of these miRNAs, miR-21 and miR-155, both up in cancer, were corroborated by independent groups. Two important limitations to this approach should be noted: 1) probe-set bias, whereby a priori probe selection defines the possible set of final observations - this effect likely explains why only two candidate miRNAs were independently corroborated; 2) normalization - all but three studies selected circulating miR-16 for endogenous normalization; however, miR-16 is predominantly derived from erythrocytes and has been shown to be particularly prone to artificial elevation by hemolysis, as high as 30-fold, which would far exceed any conceivable range of correction utilizing total red blood cell count [29]–[31].

Genome-wide platforms for high-throughput profiling of all circulating miRNA, such as an oligoarray or next generation miRNA sequencing (miR-Seq), allow agnostic/unbiased discovery of putative circulating miRNAs biomarkers as a pathway to development for breast cancer detection. In late 2010, the first such genome-wide data were reported in a pilot study by Zhao et al. [32] comparing plasma miRNA profiles of 20 breast cancer patients and 20 healthy controls on the Illumina oligoarray platform, which provides coverage of 1145 miRNAs. This resulted in identification of a short list of 26 differentially expressed plasma miRNAs (11 up and 15 down, p<0.005 sans multiple testing correction). Notably, no overlap was demonstrated between this set of 26 circulating miRNAs and previous candidate miRNA identified by qPCR-based candidate miRNA studies. Since that time, four additional genome-wide circulating miRNA studies have been reported, using miR-Seq (SOLiD or Solexa), oligoarray (Geniom, 1100 miRNAs) and TaqMan multiplex array (ABI, 446 miRNAs) platforms for agnostic discovery of candidate biomarkers in breast cancer [33]–[36]. We therefore sought to determine: 1) the degree of consensus, if any, between these genome-wide studies, and 2) to test the reproducibility of these results. We further designed our experiments to account for some possible deficiencies in current study designs that may account for some of the lack of reproduction. First, we included additional samples to allow the evaluation of any putative biomarker in post-surgical resection breast cancer cases, where the biomarker should regress to baseline, and cases of other cancers in females (colorectal and lung), to allow evaluation of specificity to breast cancer. Secondly, we filtered out miRNAs associated with blood cells that were likely to capture blood counts, which is not the intention of this study.

## Methods

### Review of Previous Genome-wide Circulating miRNA Studies

PubMed search using the terms “miRNA breast cancer” identified 732 publications, from 2003 through July 2012. Fifteen publications met the criteria of: original research comparing circulating miRNA levels between samples from breast cancer cases and healthy controls for at least one miRNA species. Studies were further categorized as genome-wide vs. probe set (qPCR). Differential expression results were harmonized across studies as simple fold-change, up or down in breast cancer, to allow efficient comparison.

### Clinical Specimens

Cases for this study were recruited from newly diagnosed breast cancer patients at University Hospitals Case Medical Center (UHCMC) and controls were recruited from individuals undergoing screening mammography at UHCMC, between 2009 and 2010. Exclusion criteria for both cases and controls included prior non-surgical treatment for any cancer or known BRCA1 or BRCA2 mutation. All participants were asked to complete a survey of breast cancer risk factors and to donate a blood sample for genetic and biomarker studies. All surveys and blood samples were obtained prior to initiation of systemic chemotherapy or hormonal therapy. Included in this study were 20 patients with blood samples collected prior to surgical resection of tumor, 20 patients with blood samples collected after tumor resection (range 6 to 62 days post) and 20 age and race matched healthy controls with negative mammography.

Additionally, we included samples in the study from 10 female subjects newly diagnosed with cancers other than breast, in order to evaluate specificity of circulating miRNA to breast cancer. Research blood samples were collected just prior to index colonoscopy from 5 female subjects recruited between 2005 and 2010, who were diagnosed with pathologically confirmed colorectal cancer as a result of the procedure, through the Case Transdisciplinary Research in Energetics and Cancer Center Colon Polyps Study [37], [38]. This study was approved by the UHCMC IRB and all study participants gave written informed consent. Research blood samples were also collected through the UHCMC Department of Thoracic Surgery solitary pulmonary nodule clinic between 2010 and 2011 from 5 females prior to scheduled surgical excision, who were found to have pathologically confirmed non-small cell lung cancer as a result of surgery, through the Genetic and Biologic Markers of Lung Cancer Study.

### Ethics Statement

This breast cancer study and the Case Transdisciplinary Research in Energetics and Cancer Center Colon Polyps Study (from which 5 colorectal cancer plasma samples were utilized) were both approved by the UHCMC institutional review board (IRB). The Genetic and Biologic Markers of Lung Cancer Study, from which 5 lung cancer samples were obtained was approved by the Case Comprehensive Cancer Center IRB. All participants in all three studies provided written informed consent.

### Sample Handling

In all instances, blood samples were processed in the same day as collection, and all samples were processed in the same lab. Whole blood was collected in standard 10 mL Vacutainer lavender-top glass tubes containing EDTA anticoagulant. Plasma was separated by centrifugation at 600 g×15 minutes at room temperature and separated into 1.0 mL aliquots which were immediately stored at −80°C until further use. All participants gave written informed consent and signed a medical record release. All studies were approved by either the UHCMC or Case Comprehensive Cancer Center institutional review board.

### RNA Isolation and miRNA Expression Profiling

Plasma samples were de-identified and lab personnel were blinded to subset status (newly diagnosed breast cancer cases, post-resection breast cancer cases, healthy controls and other lung/colon cancer) to avoid potential bias and/or batch effects. Total plasma RNA, including miRNA, was isolated using the miRNeasy kit (Qiagen #217004) according to manufacturer’s protocol, with the following modifications: 800 uL total of plasma per sample was used for extraction; each of four 200 uL aliquots was mixed with 1 µg of carrier MS2 bacteriophage RNA (Roche #10165948001) in 750 uL QIAzol reagent and incubated at room temperature (RT) for 5 minutes, followed by addition of 200 uL chloroform and incubation for additional 2 minutes; samples were centrifuged at 12,000 g for 15 minutes at 4C and then 500 uL of upper aqueous phase was carefully transferred to 1.5 volumes of 100% ethanol, which was mixed and then loaded on silica-membrane columns; columns were spun at 13,000 g for 30s at RT and this was repeated until all aliquots of an individual sample were batched on a single column; columns were washed with 700 uL of RWT buffer and spun at 13,000 g for 1 min at RT, followed by three successive washes with 500 uL RPE buffer spun at 13,000 g for 1 min at RT; after drying for 2 min at RT, elution using 50 uL of nuclease-free water was performed. A 1 ul aliquot was used for RNA fluorometric quantification (Qubit, Invitrogen) and remainder stored at −80°C until further analysis. The Illumina Human v2 Microarray (MI-101-1124, Illumina) was utilized to profile circulating levels of 1145 microRNAs. Following manufacturer’s recommendation 200 ng of isolated total RNA from each sample was used and assay performed according to supplied protocol (MicroRNA Expression Profiling Assay Guide, Illumina). The Illumina BeadArray Reader and BeadScan software were used to scan and extract raw intensity values.

### Data Analysis

Background subtraction and quantile normalization were performed using the Illumina GenomeStudio package. Expression profiles have been deposited in NCBI’s Gene Expression Omnibus (GEO) with accession number [GSE41526]. We then applied two filters to the dataset. Filter 1: circulating miRNAs derived from the cellular blood compartment may theoretically confound circulating signatures and therefore we filtered a recently published set of 140 circulating miRNA which were identified as primarily derived from the peripheral blood cellular compartment [39]. Filter 2: miRNA which were undetectable in more than 10% (N>7) of the samples after array background subtraction were filtered, because these low abundance species would have little practical reliability as candidate biomarkers.

### Statistical Analysis: miRNA Expression Associated with Breast Cancer

**Figure 1:**
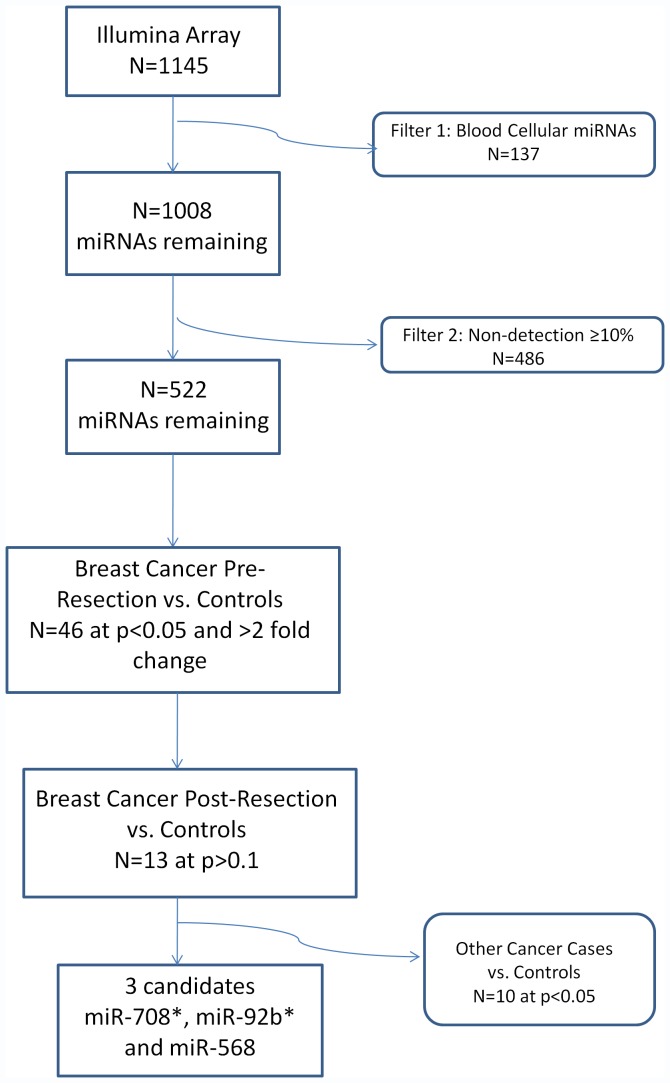
Summary of Study Design and Results.

Figure 1 provides an overview our study design and analyses performed. The statistical significance of differences in age distribution between pre-resection cases and healthy controls was calculated using a standard t-test. The significance of differences in the number of African-Americans and Caucasians between pre-resection cases and controls was done using a Fisher exact test.

We applied a standard t-test to test the association of the normalized expression values for each individual miRNA (after filtering as described above) comparing the pre-resection breast cancer cases with the healthy mammography screened controls. In those that were statistically significant, we also used a t-test to compare the controls to breast cancer patients after surgical tumor removal in order to evaluate if this miRNA regresses toward “normal” after tumor resection. Lastly, we tested the significance of the difference in expression of the controls and the “other” cancers (colorectal and lung) using another t-test to see if these miRNAs are associated with cancer in general or are breast cancer specific.

### Test of Reproducibility between Identical Platforms

We identified a single study which employed the same sample substrate (plasma) and genome-wide platform as our study (Illumina Human v2 Microarray, Zhao et al.) [32], with publically available datasets in the GEO repository. We extracted the log 2 fold-changes between the 20 cases and 20 controls from this study and corresponding unadjusted p-values for each of the 1145 miRNAs profiled on the oligoarray, using GEO2R. In order to test the global agreement among miRNAs between the two independent datasets, each of which was generated using 20 cases and 20 controls, we compared estimated log 2 fold-changes of all 1145 miRNAs to calculate a Pearson correlation coefficient and corresponding p-value. All statistics were performed using SAS 9.2.

Because this global comparison for correlation would potentially miss biologically important outlier readouts due to dilution by the non-significant findings, we performed a second test of reproducibility, the goal of which was to test for overlap in outliers between datasets. We selected the list of top candidate miRNAs in our dataset which were statistically significant using a threshold *p*-value of <0.05 with at least two-fold change between cases and controls (n = 35), and tested for differential expression in the dataset from Zhao et al. Likewise we evaluated the top candidate miRNAs from the Zhao study (n = 26), selected by identical criteria of *p*-value <0.05 and at least two-fold change between cases and controls, for differential expression in our dataset.

## Results

### Review of Previous Genome-wide Circulating miRNA Studies

Fifteen prior studies were identified which met our criterion of original research publication comparing circulating levels of at least one or more miRNA species between breast cancer cases and healthy controls. Ten of the fifteen studies were qPCR-based, using pre-selected probes to profile from 2 to 7 candidate miRNAs in the circulation, as listed in Table S1. In aggregate, 25 distinct circulating miRNAs were profiled by qPCR, using study cohorts which ranged in size from 20–102 cases and 20–85 controls. Table 1 lists the sixteen miRNAs which were found to be differentially expressed in the circulation (10 up & 6 down, in breast cancer). Two of these sixteen miRNAs were consistently identified by more than one group: miR-21 up in cancer by three groups and miR-155 up in cancer by two groups. Table S2 lists the remaining five studies which were categorized as genome-wide, using comprehensive approaches to agnostically profile circulating miRNAs for candidate biomarker discovery. Genome-wide profiling studies reported total detection of between 188 and 385 miRNAs in circulation, based on cohorts which ranged between 13–48 cases and 10–57 controls. Between these five studies, 158 candidate miRNAs were identified as differentially expressed in circulation (78 up & 80 down, in breast cancer) the complete list of which is included in Table S3. In total, only 16 of these 158 candidate miRNAs overlapped between two studies, but none overlapped in three or more studies. As shown in Table 2, inconsistent findings between studies were more common than consistent findings. Contradictory up/down results were observed for 10 of 16 overlapping miRNAs, whereas only 6 miRNAs showed consistent direction of change in two independent studies. Based on these 6 “consensus” miRNAs, overall concordance between studies was calculated as 3.8% (6/158). The six consensus miRNAs, as listed in Table 2, were: (up in breast cancer) miR-25, miR-222, miR-451, miR-497; (down in breast cancer) miR-31, miR-151-5p.

**Table 1:** qPCR Circulating miRNA Profiling Studies in Breast Cancer.

miRNA	FOLD CHANGE	SAMPLE	CASES	CTRLS	qPCR STUDY^19–28^
miR-21	up 2.5	serum	58	40	Wang
miR-21	Up	serum	102	20	Asaga
miR-21	up ∼2	serum	20	20	Wu
miR-155	up 1.6	serum	30	29	Roth
miR-155	up 3.5	serum	58	40	Wang
U6	up 1.5	serum	75	68	Appaiah
let7a	up 11.2	wholeblood	83	44	Heneghan
miR-10b	up 4	serum	30	29	Roth
miR-29a	up ∼2	serum	20	20	Wu
miR-34a	up 4.5	serum	30	29	Roth
miR-106a	up 1.9	serum	58	40	Wang
miR-126	dwn∼2	serum	58	40	Wang
miR-195	up 19.3	wholeblood	83	44	Heneghan
miR-199a	dwn∼2	serum	58	40	Wang
miR-214	up ∼5	serum	102	85	Schwarzenbach
miR-215	dwn∼2	serum	71	20	van Schooneveld
miR-299-5p	dwn∼2	serum	71	20	van Schooneveld
miR-335	dwn∼2	serum	58	40	Wang
miR-411	dwn∼2	serum	71	20	van Schooneveld

**Table 2:** Genome-Wide Circulating miRNA Profiling Studies in Breast Cancer.

STUDY^32–36^	Wu	Hu	Schrauder	Sieuwerts	Zhao
Candidate miRNAs	85	10*	25	12	26
Platform	SOLiD	Illumina GAIIx	Oligoarray	TaqMan	Oligoarray
Substrate	Serum	Serum	Whole Blood	CTCs**	Plasma
Normalization	Total reads	miR-191,484	VSN	AVG 28-miR	Quantile
N = case/ctrl	13/10	48/48	48/57	41/8	20/20
**Consistent miRNAs** *					
**miR-497**			1.8 *up*	2.9 *up*	
**miR-451**	67.8 *up*	6.5 *up*			
**miR-25**	10.7 *up*	56.0 *up*			
**miR-222**	1.9 *up*	4.8 *up*			
**miR-31**	11.1 *down*			1.4 *down*	
**miR-151-5p**	1.5 *down*				7.1 *down*
**Inconsistent miRNAs** **					
**miR-30a**	20.0 *down*	5.0 *up*			
**miR-106b**	4.4 *down*		1.7 *up*		
**miR-210**	4.4 *down*			8.1 *up*	
**let-7b**	3.9 *down*	4.1 *up*			
**miR-24**	26.7 *up*		1.8 *down*		
**miR-200c**	2.9 *up*			1.4 *down*	
**miR-155**	1.2 *down*				2.4 *down*
**miR-148a**	9.0 *up*				3.2 *down*
**miR-181a**	5.5 *up*				4.0 *down*
**miR-922**			1.7 *up*		2.4 *down*

* miRNAs consistent between at least 2 studies (fold change as cancer vs. control).

** miRNAs inconsistent between at least 2 studies (fold change as cancer vs. control).

Comparison of qPCR-based and genome-wide studies showed surprisingly little concordance. None of the 6 genome-wide consensus miRNAs were selected for study in any of ten prior qPCR profiling studies. On the other hand, the two most promising qPCR candidate miRNAs, circulating miR-21 and miR-155, which were consistently reported as 2.5 to 3.5-fold higher in breast cancer by independent groups, were directly contradicted by genome-wide results, showing that both were down in breast cancer. Using the SOLiD platform, miR-21 was reported to be 4-fold down in breast cancer, while miR-155 was reported to be 1.2 to 2.4-fold down on both the SOLiD and Illumina oligoarray platforms, respectively [32], [36].

### 2miRNA Expression Associated with Breast Cancer

We identified 20 pre-treatment female breast cancer cases (“pre-resection cases”), 20 matched female healthy volunteers (“controls”), 20 female breast cancer patients who had already undergone complete resection tumor (“post-resection cases”) and 10 female patients with either lung or colorectal cancer (“other cancer”). Table 3 shows the demographic characteristics of the patients included in this study. The pre-resection cases did not statistically significantly differ from the controls with respect to age or race.

**Table 3:** Current Study Population Characteristics.

	Controls(N = 20)	Breast Cancer Cases(N = 20)	Breast Cancer Post-Resection Cases(N = 20)	Other CancerCases (N = 10)	p (controls vs. breast cancer cases)
**Age (years), mean (SD)**	54.9 (9.1)	53.7 (9.9)	58.8 (9.7)	64.2 (5.5)	0.068
**Race, N (%)**					0.66
** African-American**	4 (20.0)	2 (10.0)	5 (25.0)	4 (40.0)	
** Caucasian**	16 (80.0)	18 (90.0)	15 (75.0)	6 (60.0)	
**Stage, N (%)**	N/A			N/A	N/A
** I**		5 (25.0)	8 (40.0)		
** II**		12 (60.0)	11 (55.0)		
** III**		1 (5.0)	1 (5.0)		
** IV**		2 (10.0)	0 (0)		
**Grade, N(%)**	N/A			N/A	N/A
** I**		4 (20.0)	4 (20.0)		
** II**		8 (40.0)	9 (45.0)		
** III**		7 (35.0)	6 (30.0)		
** Missing**		1 (5.0)	1 (5.0)		
**ER status, N (%)**	N/A			N/A	N/A
** Positive**		12 (60.0)	15 (75.0)		
** Negative**		8 (40.0)	5 (25.0)		
**PR status, N (%)**	N/A			N/A	N/A
** Positive**		10 (50.0)	15 (75.0)		
** Negative**		10 (50.0)	5 (25.0)		
**HER2/neu status, N (%)**	N/A			N/A	N/A
** Positive**		3 (15.0)	2 (10.0)		
** Negative**		17 (85.0)	16 (80.0)		
** Unknown**		0 (0)	2 (10.0)		

Filtering of 140 reported blood cellular miRNAs from 1145 miRNAs on the oligoarray, resulted in 1008 miRNAs (note not all 140 miRNAs mapped directly to a single microarray assay, resulting in the removal of 137 data points). Filtering of low abundance species, defined as undetectable in >10% of samples, further eliminated 486 miRNAs, leaving a total set of 522 miRNAs for analysis. We identified 46 miRNAs whose circulating expression were statistically significantly different between the controls and the pre-resection breast cancer cases at p<0.05 with at least 2-fold change between cases and controls (Table 4). Of these 46 miRNAs, 13 candidates met the criterion of normalizing toward baseline after surgical resection of breast cancer, (no statistically significant difference between mean levels in controls and post-resection cases at p>0.1). Ten of these 13 candidate miRNAs appeared to lack specificity to breast cancer, as evidenced by statistically significantly differences in comparisons between healthy controls vs. other cancers (p<0.05), all in the same direction (up or down regulated) as the breast cancer cases (Table 4), leaving three candidate miRNAs with evidence of specificity for breast cancer (miR-708*, miR-92b* and miR-568). Figure 1 shows a summary of our results.

**Table 4:** Top 46 differentially expressed plasma miRNAs in current study (p<0.05).

miRNA	Control mean	Breast Cancer CasesMean	Post-resection Breast Cancer Cases Mean	Other Cancers Mean	p-value (control vs. pre-resection)	p-value (control vs. post-resection)	p-value (control vs. other cancers)
**miR-1184**	606.7	2248.4	1434.8	5581.4	0.0002	0.084	0.0001
**miR-376c**	1681.5	4011.3	4204.7	5898.1	0.0003	0.0020	0.0004
miR-30b*	44.2	19.6	33.6	12.6	0.0003	0.14	0.0004
miR-200a*	20.9	126.1	81.3	330.7	0.0009	0.061	<0.0001
**miR-1295**	37.2	94.8	66.3	264.9	0.0010	0.073	0.0027
**HS_303_b**	274.8	83.4	165.5	61.9	0.0011	0.072	0.0096
**miR-34c-5p**	28.3	328.5	124.1	804.6	0.0016	0.083	<0.0001
**HS_276.1**	131.6	429.4	323.6	917.1	0.0020	0.032	<0.0001
**HS_123**	36.3	108.3	167.4	300.0	0.0020	0.22	0.0015
miR-187*	81.7	989.6	517.9	2422.7	0.0024	0.065	<0.0001
**miR-376a**	524.2	2062.3	1630.0	4388.1	0.0029	0.014	0.0030
**miR-379**	533.5	1566.0	1023.6	4098.1	0.0030	0.091	0.0013
**miR-876-5p**	24.8	12.7	16.0	11.1	0.0033	0.035	0.051
**miR-377**	431.4	1505.4	1582.6	3068.8	0.0033	0.0074	<0.0001
miR-202*	149.5	2134.3	1722.9	7324.8	0.0035	0.059	<0.0001
**miR-376b**	354.9	1159.3	1455.9	2275.8	0.0035	0.0064	<0.0001
miR-30c-2*	10.2	37.3	35.5	210.1	0.0038	0.018	0.033
**miR-1179**	176.0	1405.7	1257.4	2278.0	0.0042	0.035	<0.0001
**miR-623**	247.0	1951.5	1249.7	5334.7	0.0044	0.13	0.0003
**miR-518e**	34.2	103.6	73.1	291.3	0.0046	0.12	<0.0001
**miR-587**	17.3	36.3	26.0	283.4	0.0051	0.12	0.047
**HS_242**	145.1	1589.7	1033.1	3971.6	0.0056	0.050	0.0012
miR-193b*	235.4	84.5	246.5	68.8	0.0062	0.90	0.027
**miR-1197**	80.6	1833.8	1286.6	5082.4	0.0064	0.049	0.0003
**miR-33b**	658.7	261.0	527.0	24.2	0.0073	0.45	0.0013
miR-708*	190.5	80.8	145.0	347.2	**0.0081**	**0.30**	**0.43**
**miR-1304**	100.4	1207.5	1398.4	4469.0	0.0083	0.053	<0.0001
**miR-1261**	37.8	18.9	23.5	10.2	0.0092	0.043	0.0036
**miR-380**	151.0	2224.2	1308.4	5256.7	0.0092	0.078	<0.0001
**miR-490-3p**	35.9	17.7	46.4	5.8	0.011	0.35	0.0014
**HS_304_b**	19.2	43.7	37.6	46.4	0.011	0.049	0.0007
**miR-940**	2006.3	4695.4	3774.3	7062.9	0.012	0.099	<0.0001
**miR-1180**	238.1	697.9	723.9	2538.2	0.013	0.079	<0.0001
**miR-671-3p**	126.5	691.7	566.3	3068.5	0.014	0.032	0.0004
**miR-654-5p**	231.2	994.7	459.0	2315.0	0.014	0.22	<0.0001
miR-380*	126.6	759.4	496.9	2352.3	0.018	0.12	0.0002
**HS_149**	75.6	607.1	514.5	1857.7	0.019	0.064	0.019
miR-92b*	159.3	61.5	96.3	98.9	**0.019**	**0.156**	**0.33**
**miR-1238**	3210.5	8019.5	7376.7	12290.8	0.019	0.053	<0.0001
**miR-612**	32.8	1050.2	423.7	2872.0	0.022	0.093	0.0006
**miR-299-5p**	30.2	101.9	295.5	356.7	0.029	0.064	0.015
**miR-646**	194.9	902.0	1118.6	3544.8	0.030	0.051	0.0020
**miR-127-3p**	111.0	261.1	354.7	270.6	0.042	0.022	0.15
miR-378*	143.2	814.9	982.8	2352.0	0.043	0.097	0.00074
**miR-568**	31.2	301.2	71.0	265.1	**0.044**	**0.37**	**0.17**
**miR-202**	40.3	102.3	79.4	266.1	0.050	0.28	<0.0001

Background-subtracted, quantile normalized, average expression levels of plasma miRNAs statistically significantly different between pre-resection breast cancer patients and controls.

Comparing our results to other published studied showed a similar lack of consistency in results. Filtering of 140 blood cellular miRNAs in our study design eliminated four of the six genome-wide consensus miRNAs from our analysis (miR-25, miR-222, miR-451 and miR-151-5p). This is not surprising, as none of the prior studies incorporated provisions to adjust for blood cellular miRNA filtering. Of the two remaining consensus miRNAs, miR-497 was eliminated by pre-designated low abundance filtering for those miRNA undetectable in greater than 10% of samples. The single consensus miRNA remaining in our set of 522 miRNAs for analysis, miR-31, did not significantly differ between cases and controls in our sample (p = 0.13), although a trend to lower circulating mean levels in breast cancer was observed, consistent with the findings of other groups.

### Test of Reproducibility between Identical Platforms

**Figure 2:**
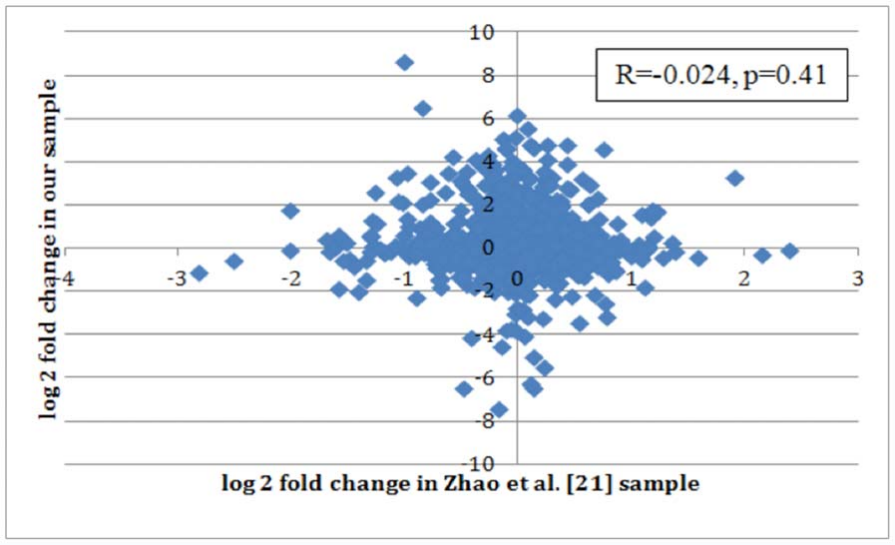
Lack of global correlation in fold change between our and Zhao et al. datasets. Correlation in fold change for all miRNAs between breast cancer cases and controls observed in our and Zhao et al. sample sets. The one agreeing data-point (up in both) is miR-431*, p = 0.15 in ours, p = 0.03 (unadjusted) in Zhao et al.

**Figure 3:**
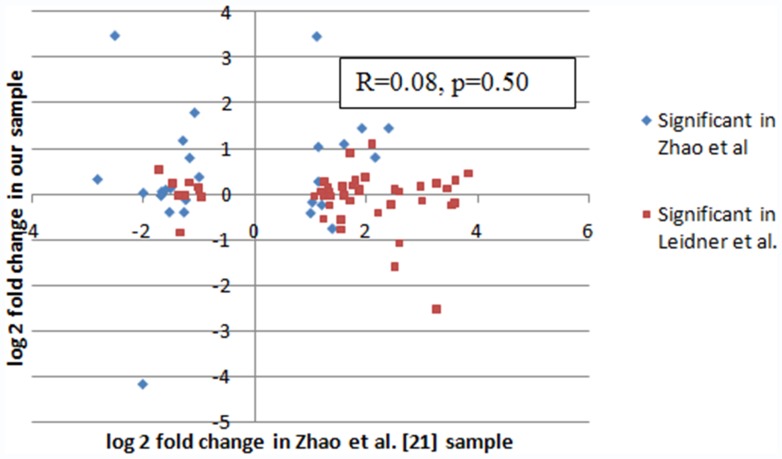
Lack of outliers correlation in fold change between our and Zhao et al. datasets miRNAs significant in one or both samples. Correlation in fold change for miRNAs significantly different between breast cancer cases and controls as observed in one, or both, studies (n = 72).

In the analysis of the correlation between estimated fold change in each of the 1145 miRNAs between our data and the Zhao et al. data, we found no evidence of an association between datasets, as shown in Figure 2. Among all 1145 miRNAs, fold changes were not correlated (R = −0.024, p = 0.41). This suggests a global lack of data agreement in the two datasets. In order to account for possibility of relevant association only in extreme data-points, we secondarily compared only the top miRNA candidates from each study. Table 5 shows the profiling results from the Zhao et al. dataset using only our top 46 miRNAs, with a comparison of the fold changes observed in both studies. Of these 46 miRNAs, the only miRNA that was statistically different between cases and controls in Zhao et al. data as well (miR-1304) was actually altered in the opposite direction. Table 6, represents the converse and shows the profiling results from our dataset using only the top 26 miRNAs identified in the study by Zhao et al. Again, the three overlapping miRNAs in this comparison (p<0.05) were altered in the opposite direction. In generating of both tables 5 and 6, identical methods of background subtraction, normalization and statistical cutoffs for significance were employed [32]. No replication of findings was observed between datasets at the top-candidates (outlier) level. Figure 3 shows the correlation in fold change observed in these two studies among those identified as significant in one. Among these 72 miRNAs, the fold changes were not correlated (R = 0.08, p = 0.50).

**Table 5:** Top 46 candidate miRNAs from current study compared in Zhao et al. dataset.

	Leidner et al.		Zhao et al.	
miRNA	Log2 FC*	P value*	Log 2 FC†	P value†
**miR-92b** *	−1.72	0.019	0.53	0.25
**miR-202**	−1.48	0.05	0.23	0.44
**miR-1197**	−1.37	0.0064	−0.02	0.97
**miR-376c**	−1.34	0.0003	−0.84	0.23
**miR-1295**	−1.24	0.001	−0.03	0.95
**miR-568**	−1.17	0.044	0.25	0.62
**miR-187** *	−1.02	0.0024	0.15	0.61
**HS_304_b**	−1.00	0.011	0.09	0.63
**miR-202** *	−0.96	0.0035	−0.06	0.41
**HS_123**	1.07	0.002	−0.05	0.80
**miR-200a** *	1.19	0.0009	0.06	0.86
**miR-380**	1.23	0.0092	−0.54	0.48
**miR-1238**	1.23	0.019	−0.04	0.83
**miR-376b**	1.25	0.0035	0.28	0.68
**HS_276.1**	1.32	0.002	0.15	0.64
**miR-34c-5p**	1.34	0.0016	−0.24	0.17
**miR-490-3p**	1.34	0.050	0.03	0.66
**miR-30c-2** *	1.35	0.0038	−0.05	0.56
**miR-1179**	1.55	0.0042	−0.78	0.40
**miR-378** *	1.55	0.043	−0.55	0.06
**miR-127-3p**	1.58	0.042	0.17	0.74
**miR-876-5p**	1.60	0.0033	−0.02	0.88
**miR-376a**	1.71	0.0029	0.91	0.21
**miR-612**	1.71	0.022	−0.15	0.87
**miR-1184**	1.75	0.0002	0.19	0.78
**miR-193b** *	1.80	0.0062	0.30	0.46
**miR-708** *	1.87	0.0081	0.05	0.92
**miR-30b** *	1.89	0.0003	0.11	0.27
**miR-671-3p**	1.98	0.014	0.38	0.51
**miR-379**	2.11	0.003	1.10	0.22
**miR-587**	2.21	0.0051	−0.41	0.47
**miR-33b**	2.45	0.0073	−0.23	0.33
**miR-654-5p**	2.51	0.014	−1.58	0.06
**miR-623**	2.51	0.043	0.10	0.91
**miR-1180**	2.59	0.013	0.05	0.95
**miR-377**	2.59	0.0033	−1.08	0.05
**miR-940**	2.98	0.012	0.17	0.30
**miR-380** *	3.00	0.018	−0.14	0.82
**HS_149**	3.00	0.019	−0.15	0.82
**miR-1304**	3.27	0.044	−2.52	0.002
**miR-299-5p**	3.27	0.029	0.24	0.69
**miR-1261**	3.45	0.0092	0.12	0.17
**miR-646**	3.54	0.03	−0.25	0.70
**HS_303_b**	3.59	0.0011	−0.20	0.25
**miR-518e**	3.60	0.0046	0.30	0.29
**HS_242**	3.84	0.0056	0.46	0.61

* Log2 fold change and p-value of our top 34 miRNAs;

† Log2 fold change and unadjusted p-value from GEO2R for differential expression between cases and controls in Zhao et al. sample.

**Table 6:** Top 26 candidate miRNAs from Zhao et al. study compared in current study dataset.

	Zhao et al.		Leidner et al.	
miRNA	Log2 FC*	P value*	Log 2 FC†	P value†
**miR-595**	2.40	0.0024	1.44	0.30
**miR-589**	2.16	0.0070	0.80	0.35
**miR-504**	1.92	0.0258	1.44	0.13
**miR-518b**	1.60	0.0353	1.09	0.31
**miR-483-5p**	1.39	0.0372	−0.76	0.11
**miR-425** *	1.20	0.0271	−0.24	0.20
**miR-493**	1.14	0.0329	1.03	0.40
**miR-187**	1.14	0.0383	0.27	0.79
**miR-431** *	1.11	0.0258	3.45	0.11
**miR-1231**	1.03	0.0239	−0.18	0.80
**solexa-9655-85**	1.00	0.0265	−0.42	0.05
**miR-668**	−1.00	0.0385	0.37	0.10
**miR-377**	−1.08	0.0485	1.78	0.0039
**miR-410**	−1.17	0.0400	0.79	0.17
**miR-922**	−1.24	0.0300	−0.13	0.55
**miR-155**	−1.27	0.0141	−0.40	0.11
**HS_169**	−1.29	0.0230	1.17	0.60
**miR-340** *	−1.51	0.0199	0.12	0.83
**HS_200**	−1.53	0.0494	−0.40	0.24
**miR-432**	−1.60	0.0476	0.09	0.81
**miR-574-3p**	−1.67	0.0379	0.05	0.79
**miR-148a**	−1.68	0.0348	−0.04	0.85
**miR-181a**	−2.00	0.0044	0.02	0.89
**miR-1275**	−2.01	0.0081	−4.17	0.10
**miR-1304**	−2.51	0.0027	3.47	0.015
**miR-151-5p**	−2.82	0.0005	0.32	0.0036

* Modified from Table 1 of Zhao et al.: differentially expressed microRNAs (P<0.05) with at least two-fold change obtained from case-versus-control comparisons in specimens of all 40 participants.

† Fold change and unadjusted p-value from our data, using quantile-normalized background-subtracted data and a t-test to test for significance between the 20 controls and 20 pre-resection breast cancer patients.

## Discussion

In our review of genome-wide circulating miRNA data in breast cancer, we observed little if any concordance between five similar and independent studies. Only six “consensus” miRNAs emerged from the total of 158 candidate miRNAs identified in prior studies, as shown in Table 2; consensus in this case, being loosely defined as any circulating miRNA with a consistent change, (up or down in breast cancer), identified by at least two independent studies. None of these six “consensus” miRNAs overlapped in three, or more, studies arguing against a biologic association. Concordance between genome-wide miRNA studies was 3.8% overall, (6/158), although this is a generous estimate. If total detectable miRNA are used as the denominator, rather than set of 158 candidate miRNAs, overall concordance falls to well below 1%. “Non-consistent” miRNAs (where a miRNA is up-trending in study A but down-trending in study B) were actually more common than consensus miRNAs, n = 10 or 6.3% (10/158), as also shown in Table 2.

Furthermore, little consistency was observed between ten earlier, non-genome-wide studies (qPCR profiling), as shown in Table 1. In particular, the findings of significantly elevated circulating miR-155 and miR-21 by qPCR in breast cancer, by independent groups, were actually contradicted by subsequent data reported by genome-wide approaches. In the serum miRNA study by Wu et al., using the SOLiD platform, and the plasma miRNA study Zhao et al., using the Illumina oligoarray, miR-155 was found to be significantly reduced by 1.2 to 2.4-fold in breast cancer vs. controls, while miR-21 was found to be significantly reduced by 4.0-fold for breast cancer vs. controls in the SOLiD/serum study by Wu et al. [32], [36]. In our study, using the Illumina oligoarray genome-wide approach, we found 46 plasma miRNAs that were significantly differentially expressed between newly diagnosed, breast cancer patients and mammography-screened controls, and identified a subset (n = 3) which showed expected normalization following tumor resection, as well as specificity for breast cancer when compared to female participants with other cancers (lung or colon). Neither miR-155 nor miR-21 were significantly differentially expressed in our study.

As a sixth genome-wide dataset, our results cast further doubt on a growing body of inconsistent data for circulating miRNA as candidate markers of breast cancer. None of the 6 “consensus” miRNA from prior genome-wide studies appeared in our list of the top 46 candidates. However, 4 of the 6 consensus miRNA were eliminated from our analysis by pre-designated filtering for the 140 circulating miRNAs predominantly derived from the blood cellular fraction and subject to high level confounding by variation in blood counts [29]–[31]; and a fifth miRNA was eliminated to due low abundance, defined in our study as failing detection in more than 10% of samples, which would likely preclude clinical applicability. The sole unfiltered consensus miRNA in our dataset, miR-31, did not reach a threshold p-value <0.05 for breast cancer in our comparison, though we did observe a downtrend for breast cancer cases, consistent in direction with the two other studies.

Public availability on GEO of original data reported by Zhao et al. [32] offered a compelling opportunity to test for reproducibility with our dataset, a comparison between two nearly identical studies. Both datasets were generated using the same platform (Illumina oligoarray), the same substrate (plasma) and identical samples size for in the discovery set (20 cases/20 controls). To our initial surprise, we were unable to demonstrate any reproducibility between datasets: neither at the global level for all 1145 miRNA theoretically detectable by the oligoarray, nor in a limited sense, by restricting comparison to the set of top significant differentially expressed miRNAs from each study (n = 46 in our dataset, n = 26 in the Zhao et al. dataset). Lack of correlation between datasets at both the global and the outlier level is represented in Figures 2 and 3.

By harmonizing data from multiple reports, we were able to demonstrate widespread inconsistencies across reported studies. Whether these inconsistencies are due to differences in study design, statistical analyses, shortcomings of current technology, or other factors, remains to be answered. It may well be, that technical variance introduced by non-uniform sample handling and processing, the effects of long-term storage of archival samples or contamination by miRNA from the blood cellular fraction are all contributing to the generation of artifact, resulting non-reproducible results, as we have demonstrated. Growing caution regarding circulating miRNA discovery is being advanced by several recent methodological investigations which demonstrate, in particular, the high level of confounding introduced by blood cellular components [29, 31. 40]. Furthermore, a recent study illustrated a high level of difference in miRNA profiles between serum and plasma from the same individual [41], highlighting the fact that choice of substrate may be an important design consideration for which there is no current standard. Schrauder et al., in one of the five prior studies we reviewed in our analysis, first noted a lack of reproducibility comparing to a single previously published genome-wide study at the time (Zhao et al.), raising early concerns over technical variance as a source of confounding [34]. The results of our current analysis substantially corroborate these concerns, and lead us to the discouraging conclusion that initial enthusiasm for circulating miRNA as an approach to screening and detection of breast cancer ought to be dampened.

Ours is the first genome-wide study to pre-designate filtering of miRNAs originating from blood cellular components, in order to narrow the focus of discovery to tumor-specific circulating miRNA candidates. Although this is a major strength of our study, we did base our filtering on a single study investigating the expression of miRNAs in blood cells. Independent replications of this earlier study would help create a more robust list of blood cellular expressed miRNAs.

Ours is also the first study to include a separate cohort of post-resection samples, which we hypothesized would show a normalization to baseline levels for putative circulating miRNA markers that are truly related to breast cancer. In our study we utilized samples from 20 breast cancer patients prior to resection and 20 different breast cancer patients after tumor resection. Although not available for this study, future studies using samples from the same patients collected before and after surgical resection would be most useful for identifying miRNAs that regress to normal with removal of the tumor. However, we feel that using a sample of post-resection patients achieved our goal of identifying the overall levels of miRNAs in a typical breast cancer patient after tumor resection.

The fourth cohort included in our study, females with lung or colon cancer, was intended to preliminary ask whether a putative circulating miRNA was related to cancer per se, or breast cancer specifically. A possibility exists in our study design that the healthy control population was not cancer-free. These samples were collected from women being screened for breast cancer, and subsequently reported to have a negative mammogram, but it was not a requirement of the study that they were screened for other cancers; while very unlikely, they may have another undiagnosed cancer. A substantial limitation to this and other studies of circulating miRNAs, which may in part explain the lack of reproducibility we have demonstrated, is a lack of standardization in the field. It is not clear how circulating miRNA levels vary by sample collection protocols, handling/storage and isolation techniques [40], [42]. Just as importantly, there is no current consensus on the best way to normalize circulating measurements, because a universal housekeeping miRNA has yet to be identified in the circulation [35]. This fundamental limitation is likely a significant source inter-individual variance [43], [44].

In our study design, we chose to filter out “low abundance” miRNAs, or those that were not found in more than 10% of the samples. Our main reason for filtering these out was that low expression values are within the sensitivity of the microarray and we may be finding array artifacts. However, in this design we may also filter out miRNAs that are only found in breast cancer patients or controls, which may turn out to be an excellent discriminator. To see if this was indeed the case, we went back to the 486 miRNAs we filtered due to low abundance. Of these, 97 had an expression <0 in more than 75% of both pre-resection cases and controls. Of the remaining 389, 205 had an average expression, among those where the expression was >0 of 100 or less. Given the average overall expression was >1000, we feel that these low quantile-normalized background-subtracted expression values are within the margin of error of the array and normalization methods and may not be expressed in most of the other samples as well. Of the remaining 184, 23 were statistically significantly differentially expressed between pre-resection cases and controls (p<0.05, Table S4). However, none of the 23 overlapped with any previous study either, and therefore our conclusions are not changed. Future studies using more sensitive methods, such as qPCR, may find that these are indeed able to offer some information with regards to likely breast cancer status.

In conclusion, our additional data and comprehensive review of the literature suggests that there is still substantial work that would need to be done in order to identify an individual circulating miRNA, or set of circulating miRNAs, that could be used to identify women who have breast cancer. Further work needs to be done in order to develop standards for circulating miRNA studies, including sample preparation standards, controls for circulating blood cellular components and normalization of measured values. Although a number of studies have reported positive findings, the near complete lack of concordance suggests that, at this time, the utility of miRNAs for breast cancer detection is still questionable.

## Supporting Information

Table S1 **Comparison of prior candidate qPCR-based studies.** Comparison of the ten pre-selected candidate miRNA studies.
(DOCX)

Table S2 **Comparison of prior genome-wide miRNA profiling studies.** Comparison of the five prior studies that used comprehensive approaches to agnostically profile circulating miRNAs for candidate biomarker discovery in breast cancer.
(DOCX)

Table S3 **Overlap in results of earlier genome-wide studies.** miRNAs that were identified in two or more of the five genome-wide studies.
(DOCX)

Table S4 **List of low abundance miRNAs that were statistically significantly associated with breast cancer.** List of all miRNAs that were filtered out due to low abundance but, had they not been filtered, would be statistically significantly differentially expressed between pre-resection cases and controls.
(DOCX)
