# Supplementary material for: Dampening Enthusiasm for Circulating MicroRNA in Breast Cancer
Source: PLoS One. 2013 Mar 5;8(3):e57841. doi: 10.1371/journal.pone.0057841 (PMC3589476; doi:10.1371/journal.pone.0057841)
Supplement: Table S1 — Comparison of prior candidate qPCR-based studies. Comparison of the ten pre-selected candidate miRNA studies. (DOCX) [file pone.0057841.s001.docx]

**Table S1**

| **qPCR STUDY** | **GROUP** | **YEAR** | **SOURCE** | **CASES** | **CTRLS** | **qPCR PROBE SET** | **NORMALIZATION** | **CANDIDATE miRNAs** | **NOTES** |
| --- | --- | --- | --- | --- | --- | --- | --- | --- | --- |
| Zhu | North Dakota, USA | 2009 | serum | 13 | 8 | (miR-16,145,155) | 18s rRNA | none |  |
|  |  |  |  |  |  |  |  |  |  |
| Heneghan | Galway, Ireland | 2010 | whole blood | 83 | 44 | (10b,21,145,155,195,let-7a,16) | miR-16 | miR-195 |  |
|  |  |  |  |  |  |  |  | let-7a |  |
|  |  |  |  |  |  |  |  |  |  |
| Heneghan | Galway, Ireland | 2010 | whole blood | 83 | 63 | (10b,21,145,155,195,let-7a,16) | miR-16 | miR-195 |  |
|  |  |  |  |  |  |  |  |  |  |
| Roth | Hamburg, Germany | 2010 | serum | 30 | 29 | (10b,34a,141,155,16) | miR-16 | miR-155 |  |
|  |  |  |  |  |  |  |  | miR-10b |  |
|  |  |  |  |  |  |  |  | miR-34a |  |
|  |  |  |  |  |  |  |  |  |  |
| Wang | Hangzhou, China | 2010 | serum | 58 | 40 | (21,106a,126,155,199a,335,16) | miR-16 | miR-21 |  |
|  |  |  |  |  |  |  |  | miR-106a |  |
|  |  |  |  |  |  |  |  | miR-155 |  |
|  |  |  |  |  |  |  |  | miR-126 |  |
|  |  |  |  |  |  |  |  | miR-199a |  |
|  |  |  |  |  |  |  |  | miR-335 |  |
|  |  |  |  |  |  |  |  |  |  |
| Appaiah | Indiana, USA | 2011 | serum | 33 | 115 | (16,21,155,let-7f,U6,5S,U44) | SNORD44 | U6 | U6, 5S and miR-16 inaqeduate for normalization |
|  |  |  |  |  |  |  |  |  |  |
| Asaga | California, USA | 2011 | serum | 102 | 20 | (16,21) | miR-16 | miR-21 | direct serum qPCR using Tween20 |
|  |  |  |  |  |  |  |  |  |  |
| Wu | Nanjing, China | 2011 | serum | 20 | 20 | (29a,23a,23b,192,21,U6) | RNU6B | miR-21 |  |
|  |  |  |  |  |  |  |  | miR-29a |  |
|  |  |  |  |  |  |  |  |  |  |
| van Schooneveld | Antwerp, Belgium | 2012 | serum | 75 | 20 | (215,299-5p,411,452,16) | miR-16 | miR-411 |  |
|  |  |  |  |  |  |  |  | miR-215 |  |
|  |  |  |  |  |  |  |  | miR-299-5p |  |
|  |  |  |  |  |  |  |  |  |  |
| Schwarzenbach | Hamburg, Germany | 2012 | serum | 102 | 85 | (19a,20a,21,214,16) | miR-16 | miR-214 |  |
