# Supplementary material for: Dampening Enthusiasm for Circulating MicroRNA in Breast Cancer
Source: PLoS One. 2013 Mar 5;8(3):e57841. doi: 10.1371/journal.pone.0057841 (PMC3589476; doi:10.1371/journal.pone.0057841)
Supplement: Table S2 — Comparison of prior genome-wide miRNA profiling studies. Comparison of the five prior studies that used comprehensive approaches to agnostically profile circulating miRNAs for candidate biomarker discovery in breast cancer. (DOCX) [file pone.0057841.s002.docx]

**Table S2**

| **GENOME-WIDE STUDY** | **GROUP** | **YEAR** | **SOURCE** | **CASES** | **CTRLS** | **PLATFORM**  **(N=total miRNAs)** | **Number ANALYZED** | **NORMALIZATION** | **CANDIDATE miRNAs** |
| --- | --- | --- | --- | --- | --- | --- | --- | --- | --- |
| Zhao | Roswell Park, USA | 2010 | plasma | 20 | 20 | Oligoarray, Illumina (1145) | 266 | Quantile | 11 up / 15 down |
| Sieuwerts | Belgium/Holland | 2011 | CellSearch (EpCAM) | 41 | 8 | TaqMan multiplex, ABI (446) | 253 | Median of 28 miRNAs | 9 up / 3 down |
| Schrauder | Erlangen, Germany | 2012 | whole blood | 48 | 57 | Oligoarray, Geniom (1100) | 240 | VSN | 10 up / 15 down |
| Hu | Nanjing, China | 2012 | serum | 48 | 48 | Illumina GAIIx | 385 | miR-191,484 | 10 up / NR* |
| Wu | Nanjing, China | 2012 | serum | 13 | 10 | SOLiD | 188 | Total Read Count | 38 up / 47 down |
|  |  |  |  |  |  |  |  |  |  |
| *Reported only top 10 upregulated circulating miRNAs in breast cancer | | | |  |  |  |  |  |  |
