# Supplementary material for: Dampening Enthusiasm for Circulating MicroRNA in Breast Cancer
Source: PLoS One. 2013 Mar 5;8(3):e57841. doi: 10.1371/journal.pone.0057841 (PMC3589476; doi:10.1371/journal.pone.0057841)
Supplement: Table S3 — Overlap in results of earlier genome-wide studies. miRNAs that were identified in two or more of the five genome-wide studies. (DOCX) [file pone.0057841.s003.docx]

**Table S3**

| **UP in BRCA miRNA** | **FOLD CHANGE (up)** | **DOWN in BRCA miRNA** | **FOLD CHANGE (down)** |
| --- | --- | --- | --- |
|  |  |  |  |
| Zhao |  | Zhao |  |
| **595** | 5.3 | **668** | 2.0 |
| **589** | 4.5 | **377** | 2.1 |
| **504** | 3.8 | **410** | 2.3 |
| **518b** | 3.0 | **922** | 2.4 |
| **483-5p** | 2.6 | **155** | 2.4 |
| **425*** | 2.3 | **HS_169** | 2.5 |
| **493** | 2.2 | **340*** | 2.9 |
| **187** | 2.2 | **HS_200** | 2.9 |
| **431*** | 2.2 | **432** | 3.0 |
| **1231** | 2.0 | **574-3p** | 3.2 |
| **sol_9655-85** | 2.0 | **148a** | 3.2 |
|  |  | **181a** | 4.0 |
| Sieuwerts |  | **1275** | 4.0 |
| **379** | 4.8 | **1304** | 5.7 |
| **210** | 8.1 | **151-5p** | 7.1 |
| **424** | 3.1 |  |  |
| **452** | 4.7 | Sieuwerts |  |
| **184** | 6.2 | **31** | 1.4 |
| **497** | 2.9 | **205** | 1.1 |
| **183** | 3.5 | **200c** | 1.4 |
| **565** | 2.5 |  |  |
| **RNU19** | 1.6 | Schrauder |  |
|  |  | **718** | 3.1 |
| Schrauder |  | **625*** | 3.1 |
| **4306** | 2.1 | **1471** | 2.2 |
| **202** | 2.0 | **193a-3p** | 2.2 |
| **4257** | 2.0 | **182** | 2.1 |
| **1323** | 1.9 | **1915** | 2.0 |
| **335** | 1.9 | **564** | 1.9 |
| **497** | 1.8 | **107** | 1.8 |
| **106b** | 1.7 | **2355** | 1.8 |
| **922** | 1.7 | **3186-3p** | 1.8 |
| **516b** | 1.6 | **24** | 1.8 |
| **let7a*** | 1.4 | **3130-3p** | 1.8 |
|  |  | **526a** | 1.8 |
| Hu |  | **1469** | 1.7 |
| **let7b** | 4.1 | **874** | 1.7 |
| **151-3p** | 38.5 |  |  |
| **16** | 3.0 | Wu |  |
| **222** | 4.8 | **let7a** | 4.5 |
| **25** | 56.0 | **let7b** | 3.8 |
| **30a** | 5.0 | **let7c** | 2.9 |
| **324-3p** | 5.6 | **let7f** | 2.6 |
| **339-3p** | 14.8 | **let7g** | 3.1 |
| **451** | 6.5 | **let7i** | 3.3 |
| **486** | 10.2 | **100** | 3.2 |
|  |  | **106b** | 4.3 |
| Wu |  | **10b** | 1.1 |
| **103** | 1.6 | **125b** | 1.5 |
| **10a** | 1.6 | **126** | 33.3 |
| **124** | 8.1 | **126*** | 14.3 |
| **125a-5p** | 2.1 | **1274b** | 2.3 |
| **148a** | 9.0 | **130b** | 1.1 |
| **150** | 3.4 | **140-3p** | 3.0 |
| **181a** | 5.5 | **141** | 1.2 |
| **181b** | 4.4 | **151-5p** | 1.5 |
| **183** | 1.0 | **155** | 1.2 |
| **185** | 6.1 | **17** | 2.2 |
| **192** | 85.5 | **17*** | 1.5 |
| **193b** | 1.6 | **181c** | 25.0 |
| **194** | 4.0 | **191** | 5.9 |
| **196b** | 1.1 | **193a-5p** | 2.1 |
| **200b** | 6.7 | **1975** | 2.1 |
| **200c** | 2.9 | **19b** | 33.3 |
| **22*** | 1.8 | **200a** | 2.4 |
| **222** | 1.9 | **20a** | 33.3 |
| **223** | 2.4 | **21** | 4.0 |
| **224** | 1.3 | **210** | 4.3 |
| **23a** | 4.0 | **221** | 5.3 |
| **23b** | 1.5 | **30a** | 20.0 |
| **24** | 26.7 | **30b** | 4.5 |
| **25** | 10.7 | **30c** | 1.6 |
| **26a** | 4.0 | **30d** | 8.3 |
| **27a** | 1.5 | **31** | 11.1 |
| **27b** | 7.0 | **324-5p** | 3.7 |
| **29a** | 4.4 | **342-3p** | 2.0 |
| **29b** | 1.3 | **345** | 3.0 |
| **29c** | 3.5 | **34a** | 5.6 |
| **30e** | 1.5 | **409-3p** | 5.3 |
| **320a** | 2.2 | **455-3p** | 1.1 |
| **361-5p** | 1.2 | **532-5p** | 1.5 |
| **378** | 1.2 | **574-5p** | 1.2 |
| **429** | 3.3 | **7** | 3.7 |
| **4305** | 7.1 | **877** | 1.3 |
| **451** | 67.8 | **93** | 25.0 |
| **92a** | 2.2 | **99a** | 3.8 |
|  |  |  |  |
| Leidner |  | Leidner |  |
| **587** | 2.1 | **HS_303_b** | 3.3 |
| **HS_304_b** | 2.3 | **193b*** | 2.8 |
| **940** | 2.3 | **92b*** | 2.6 |
| **127-3p** | 2.4 | **33b** | 2.5 |
| **376c** | 2.4 | **708*** | 2.4 |
| **1238** | 2.5 | **30b*** | 2.3 |
| **202** | 2.5 | **490-3p** | 2.0 |
| **1295** | 2.5 | **1261** | 2.0 |
| **1180** | 2.9 | **876-5p** | 2.0 |
| **379** | 2.9 |  |  |
| **HS_123** | 3.0 | Hu | N/R |
| **518e** | 3.0 |  |  |
| **HS_276.1** | 3.3 |  |  |
| **376b** | 3.3 |  |  |
| **299-5p** | 3.4 |  |  |
| **377** | 3.5 |  |  |
| **30c-2*** | 3.7 |  |  |
| **1184** | 3.7 |  |  |
| **376a** | 3.9 |  |  |
| **654-5p** | 4.3 |  |  |
| **646** | 4.6 |  |  |
| **671-3p** | 5.5 |  |  |
| **378*** | 5.7 |  |  |
| **380*** | 6.0 |  |  |
| **200a*** | 6.0 |  |  |
| **623** | 7.9 |  |  |
| **1179** | 8.0 |  |  |
| **HS_149** | 8.0 |  |  |
| **568** | 9.7 |  |  |
| **HS_242** | 11.0 |  |  |
| **34c-5p** | 11.6 |  |  |
| **1304** | 12.0 |  |  |
| **187*** | 12.1 |  |  |
| **202*** | 14.3 |  |  |
| **380** | 14.7 |  |  |
| **1197** | 22.7 |  |  |
| **612** | 32.0 |  |  |
