# Supplementary material for: Dampening Enthusiasm for Circulating MicroRNA in Breast Cancer
Source: PLoS One. 2013 Mar 5;8(3):e57841. doi: 10.1371/journal.pone.0057841 (PMC3589476; doi:10.1371/journal.pone.0057841)
Supplement: Table S4 — List of low abundance miRNAs that were statistically significantly associated with breast cancer. List of all miRNAs that were filtered out due to low abundance but, had they not been filtered, would be statistically significantly differentially expressed between pre-resection cases and controls. (DOCX) [file pone.0057841.s004.docx]

**Table S4**

| **miRNA** | **Control mean** | **Breast Cancer Cases Mean** | **Post-resection Breast Cancer Cases Mean** | **Other Cancers Mean** | **p-value (control vs. pre-resection)** | **p-value (control vs. post-resection)** | **p-value (control vs. other cancers)** |
| --- | --- | --- | --- | --- | --- | --- | --- |
| hsa-miR-1181 | 56.5 | 1121.5 | 317.7 | 3303.8 | 0.0037 | 0.1511 | <0.0001 |
| hsa-miR-518f | 44.0 | 890.4 | 586.5 | 2784.7 | 0.0048 | 0.0937 | <0.0001 |
| hsa-miR-645 | 122.6 | 70.4 | 117.9 | 48.4 | 0.0051 | 0.8535 | 0.0546 |
| hsa-miR-610 | 132.4 | 50.5 | 98.1 | -27.6 | 0.0053 | 0.2509 | <0.0001 |
| HS_228.1 | 47.6 | 945.4 | 575.4 | 3619.9 | 0.0060 | 0.0371 | 0.0029 |
| hsa-miR-1183 | 44.0 | 1415.7 | 1164.2 | 4012.6 | 0.0071 | 0.1160 | <0.0001 |
| HS_257 | 545.1 | 260.3 | 762.7 | 17.2 | 0.0080 | 0.4260 | 0.0001 |
| hsa-miR-520e | 112.6 | 1201.6 | 899.0 | 2983.2 | 0.0108 | 0.1057 | 0.0012 |
| hsa-miR-32 | 1179.0 | 586.6 | 906.0 | 52.7 | 0.0204 | 0.2883 | 0.0005 |
| hsa-miR-501-5p | 627.8 | 338.2 | 480.6 | -20.2 | 0.0214 | 0.2352 | 0.0001 |
| solexa-5169-164 | 392.0 | 214.5 | 427.3 | 69.7 | 0.0306 | 0.7870 | 0.0027 |
| hsa-let-7f-1* | 457.5 | 285.2 | 389.6 | 18.3 | 0.0317 | 0.3528 | <0.0001 |
| hsa-miR-124a:9.1 | 25.4 | 541.4 | 307.1 | 1923.5 | 0.0319 | 0.1108 | 0.0008 |
| hsa-miR-154* | 99.7 | 253.8 | 237.3 | 18.1 | 0.0329 | 0.0559 | 0.0040 |
| hsa-miR-30a* | 2185.1 | 1309.8 | 1596.3 | 54.1 | 0.0340 | 0.1347 | <0.0001 |
| hsa-miR-944 | 35.1 | 374.6 | 739.7 | 2945.0 | 0.0393 | 0.0472 | 0.0001 |
| hsa-miR-487a | 104.4 | 276.9 | 356.1 | 54.5 | 0.0399 | 0.1282 | 0.4106 |
| hsa-miR-1224-5p | 522.9 | 237.8 | 319.3 | 519.7 | 0.0409 | 0.1667 | 0.9897 |
| hsa-miR-219-5p | 68.8 | 815.1 | 377.4 | 811.7 | 0.0428 | 0.0833 | 0.0311 |
| hsa-miR-452 | 101.5 | 241.0 | 122.1 | 84.8 | 0.0432 | 0.4653 | 0.6694 |
| HS_127.1 | 176.6 | 92.5 | 226.3 | 155.0 | 0.0454 | 0.6579 | 0.8520 |
| hsa-let-7g* | 111.1 | 45.0 | 123.3 | 26.2 | 0.0186 | 0.8573 | 0.0270 |
